# Supplementary material for: Conformation and dynamics of soluble repetitive domain elucidates the initial β-sheet formation of spider silk
Source: Nat Commun. 2018 May 29;9:2121. doi: 10.1038/s41467-018-04570-5 (PMC5974136; doi:10.1038/s41467-018-04570-5)
Supplement: Supplementary file 1 — Supplementary Information [file 41467_2018_4570_MOESM1_ESM.pdf]

Supplementary Information

Conformation and dynamics of soluble repetitive domain elucidates the initial β-sheet formation of spider silk

Oktaviani et al

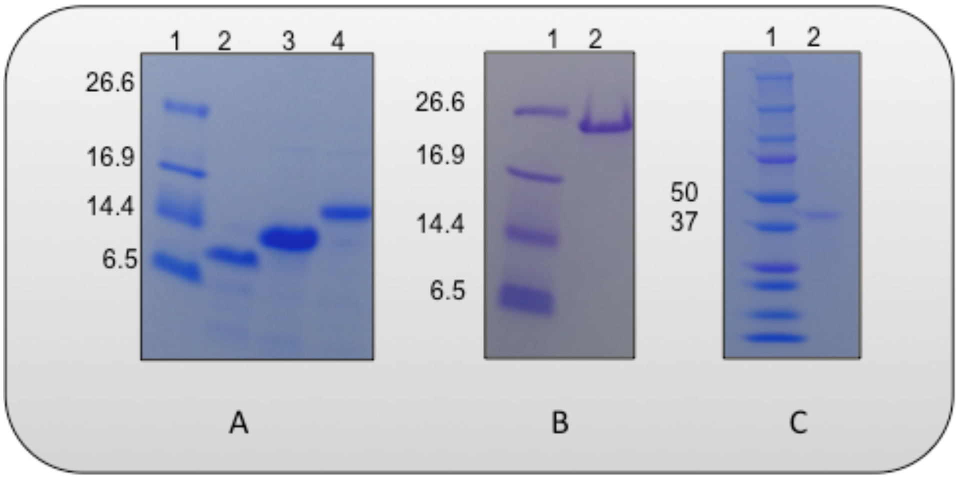

Supplementary Figure 1: SDS-PAGE profile of the purified repetitive domains used in this study. (A) Marker, monomer (7.8 kDa), dimer (10.5 kDa) and trimer (13.1 kDa) are shown in Tris-tricine 10–20% SDS-PAGE Lanes 1A, 2A, 3A and 4A, respectively. (B) Hexamer is presented using 4–20% SDS-PAGE. Lanes 1B and 2B are the marker and hexamer (20.9 kDa), respectively. (C) Migration of the 15-mer (46.2 kDa) is shown using 4–15% SDS-PAGE. Lanes 1C and 2C are the marker and 15-mer, respectively.

Supplementary Discussion

To obtain highly concentrated samples, the His-tag domain was not cleaved. The presence of the His-tag domain signals was clearly observed in the monomer spectrum, and as the number of repetitive domains increased, the His-tag domain signals decreased relative to the repetitive domain signals (**Supplementary Fig. 2B**). Therefore, in the 2D ^{1}H-^{15}N HSQC spectrum of the 15-mer, only repetitive domain signals were detectable (**Fig. 2A**). To determine whether the His-tag domain and cloning site (Thr-Ser) affected the repetitive domain signals, the spectra of the monomer with and without the His-tag domain as well as the cloning site were compared (**Supplementary Fig. 2C**). The spectrum of the monomer with the His-tag domain and the cloning site overlaid very well with that of the monomer without the His-tag and cloning site, indicating that the presence of the His-tag and cloning site did not affect the conformation of the repetitive domain. The difference in chemical shifts between those spectra was only observed for the N-terminal and C-terminal amino acids. Slight differences in chemical shift positions were found on one residue after the N-terminal region and one residue before the C-terminal region (**Supplementary Fig. 2C**).

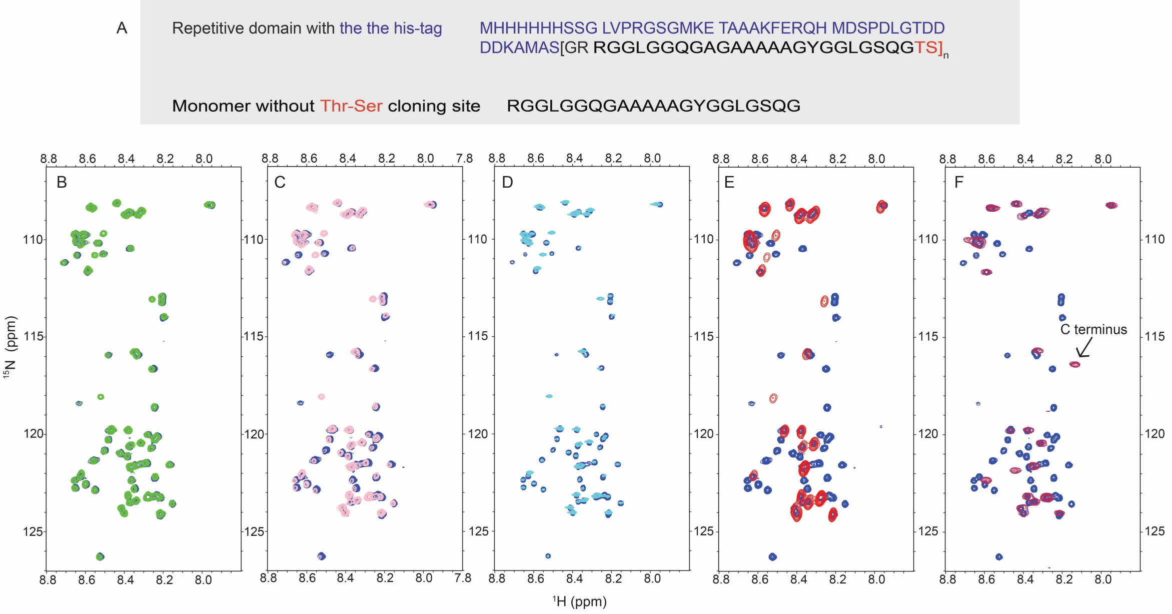

Supplementary Figure 2: Different oligomers of the repetitive domain and the influence of the His-tag domain and Thr-Ser cloning site on the spectrum of the repetitive domain. (A) Amino acid sequences of the repetitive domain (black) with the His-tag domain (blue) (upper) and amino acid sequences of the monomer without the His-tag and Thr-Ser (red) cloning site (bottom). For the monomer, dimer, trimer, hexamer and 15-mer, *n* is 1, 2, 3, 6, and 15, respectively. (B) 2D ^{1}H-^{15}N HSQC overlay of the monomer (blue) and dimer (green). (C) 2D ^{1}H-^{15}N HSQC overlay of the monomer (blue) and trimer (pink). (D) 2D ^{1}H-^{15}N HSQC overlay of the monomer (blue) and hexamer (cyan) (E) 2D ^{1}H-^{15}N HSQC overlay of the monomer (blue) and 15-mer (red). (F) 2D ^{1}H-^{15}N HSQC overlay of the monomer with (blue) and without (maroon) the His-tag. All spectra were recorded at pH 7 and 10°C. The C-terminus is indicated. The additional peak (8.414 ppm; and 121.895 ppm) is likely caused by the amide proton and amide nitrogen signal of L4. The monomer spectra with and without the His-tag and Thr-Ser cloning site overlay very well, suggesting that the His-tag and Thr-Ser cloning site do not affect the conformation of the repetitive domain.

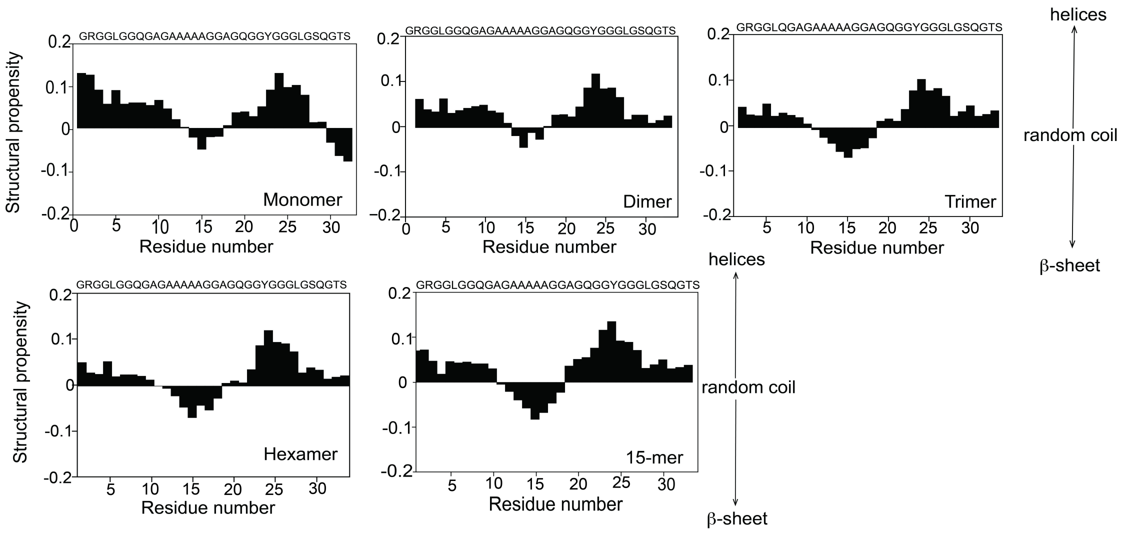

Supplementary Figure 3: Structural propensity of the recombinant repetitive domain of different lengths at pH 7 and 10°C. (A) Monomer (B), dimer (C), trimer (D) hexamer, and (E) 15-mer based on backbone chemical shifts. The structural propensity was calculated using the neighbor corrected structural propensity calculator (ncSPC).

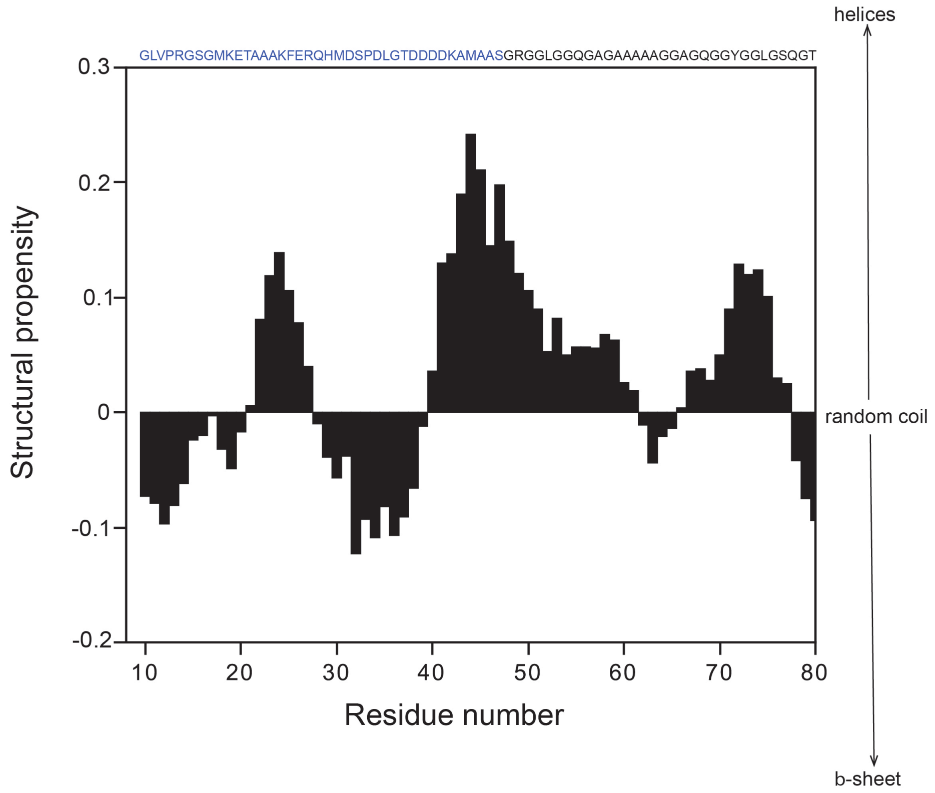

Supplementary Figure 4: Structural propensity of the monomer with the His-tag domain at pH 7 and 10°C. The corresponding amino acid sequences of the monomer with the His-tag domain are shown below. The His-tag domain is shown in blue, and the monomer is shown in black. Signals from the first 9 residues of the His-tag region, which are MHHHHHHSS, cannot be detected.

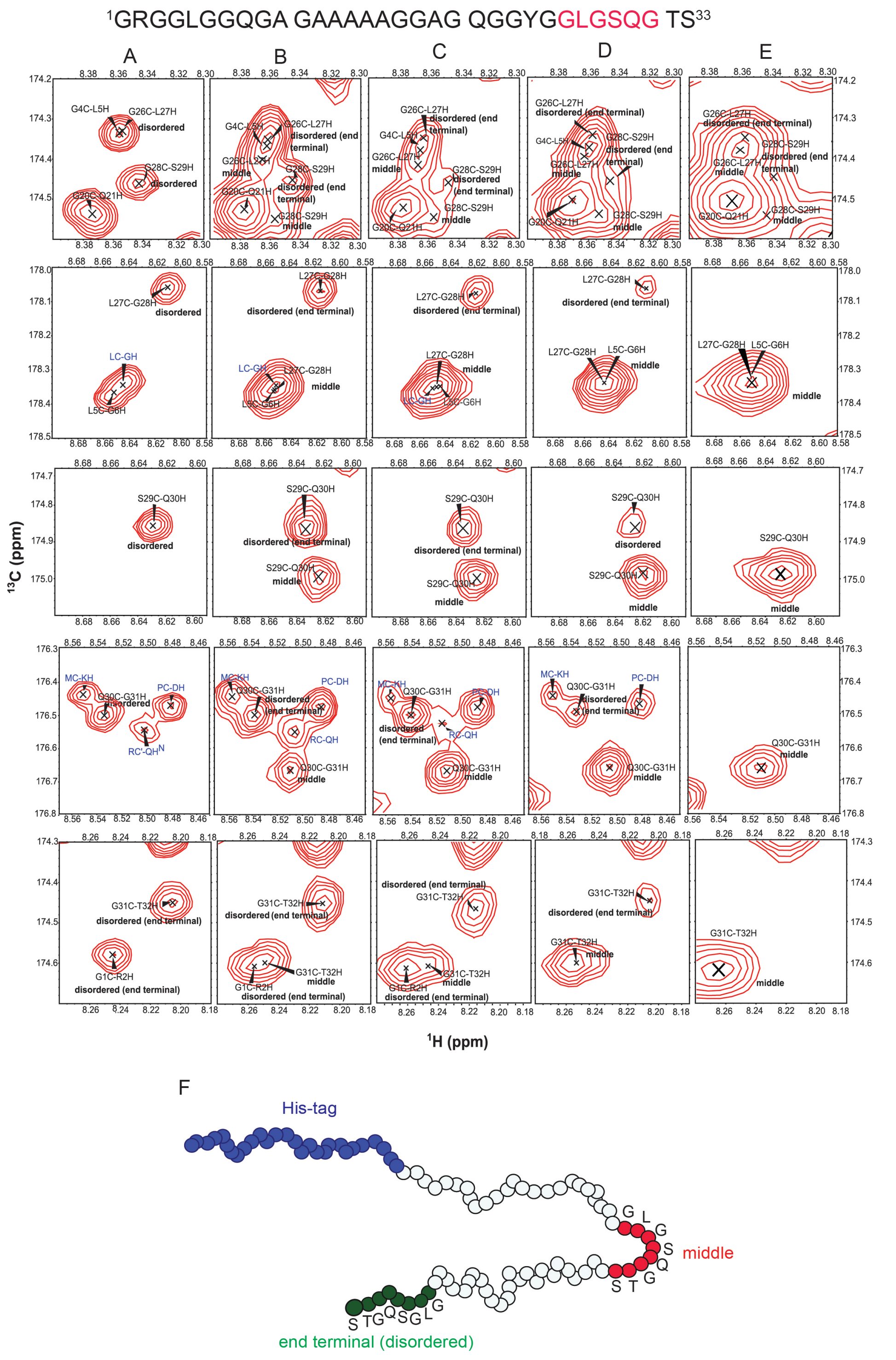

Supplementary Figure 5: When another repetitive domain is added, the carbonyl chemical shift of the GLGSGQ (written in red, above) residues changes relative to that of the monomer. Carbonyl chemical shifts of T32, S33, G1 and R2 also change but are not shown in this figure. (A) Monomer, (B) dimer, (C) trimer, (D) hexamer, and (E) 15-mer. (F) illustration of the position of GLGSQG residues in the middle (indicated in red) and in the end terminal (indicated in green) of repetitive domain (dimer). Peaks corresponding to the His-tag domain are indicated in blue.

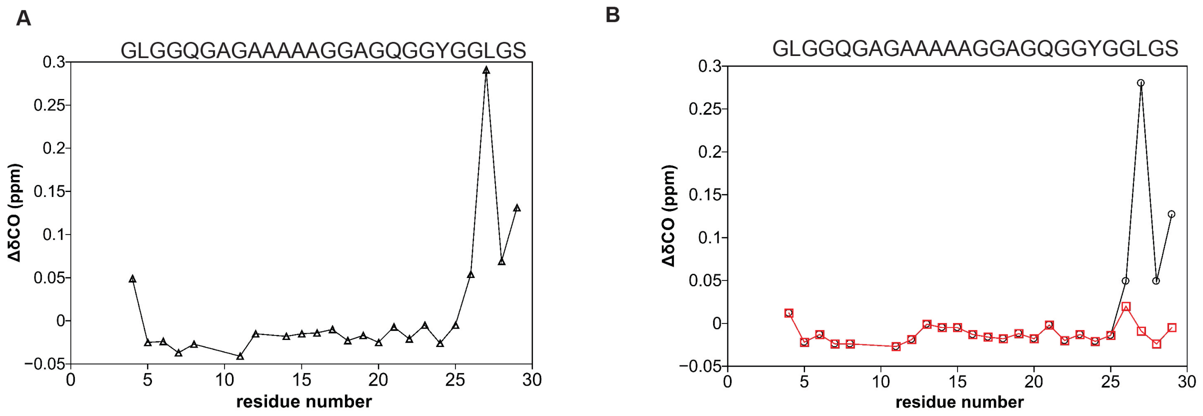

Supplementary Figure 6: Carbonyl chemical shift differences between the 15-mer and monomer and between the 15-mer and dimer as function of residue number. (A) Carbonyl chemical shift difference between the 15-mer and monomer as function of residue number. This plot does not include ^{1}GRG^{2} and ^{30}QGT^{32}, which are located at the interface between the repetitive domain with the His-tag and interface between one repetitive domain and another repetitive domain, respectively. The positive deviation suggests that the helicity increases in the 15-mer relative to the monomer. The presence of extensive carbonyl chemical shift differences between the 15-mer and monomer, which are found beyond the interface of repetitive domain, strongly indicates that the 15-mer is not a completely random coil protein. (B) Carbonyl chemical shift differences between the 15-mer and dimer in the middle region (indicated in red square) and between the 15-mer and dimer in the end terminal region (indicated in black circle) as function of residue number. As shown in Supplementary Figure 5, the backbone carbonyl of the dimer in GLGSQG region consist of 2 peaks, which correspond to GLGSQG in the middle and end terminal of repetitive domain. The chemical shift difference between backbone carbonyl of the 15-mer and dimer in the middle is close to zero, except for Arg because this residue is in the interface between repetitive domain and His-tag. In contrast, the chemical shift difference between backbone carbonyl of the 15-mer and dimer in the end terminal is similar to chemical shift difference between the 15-mer and monomer.

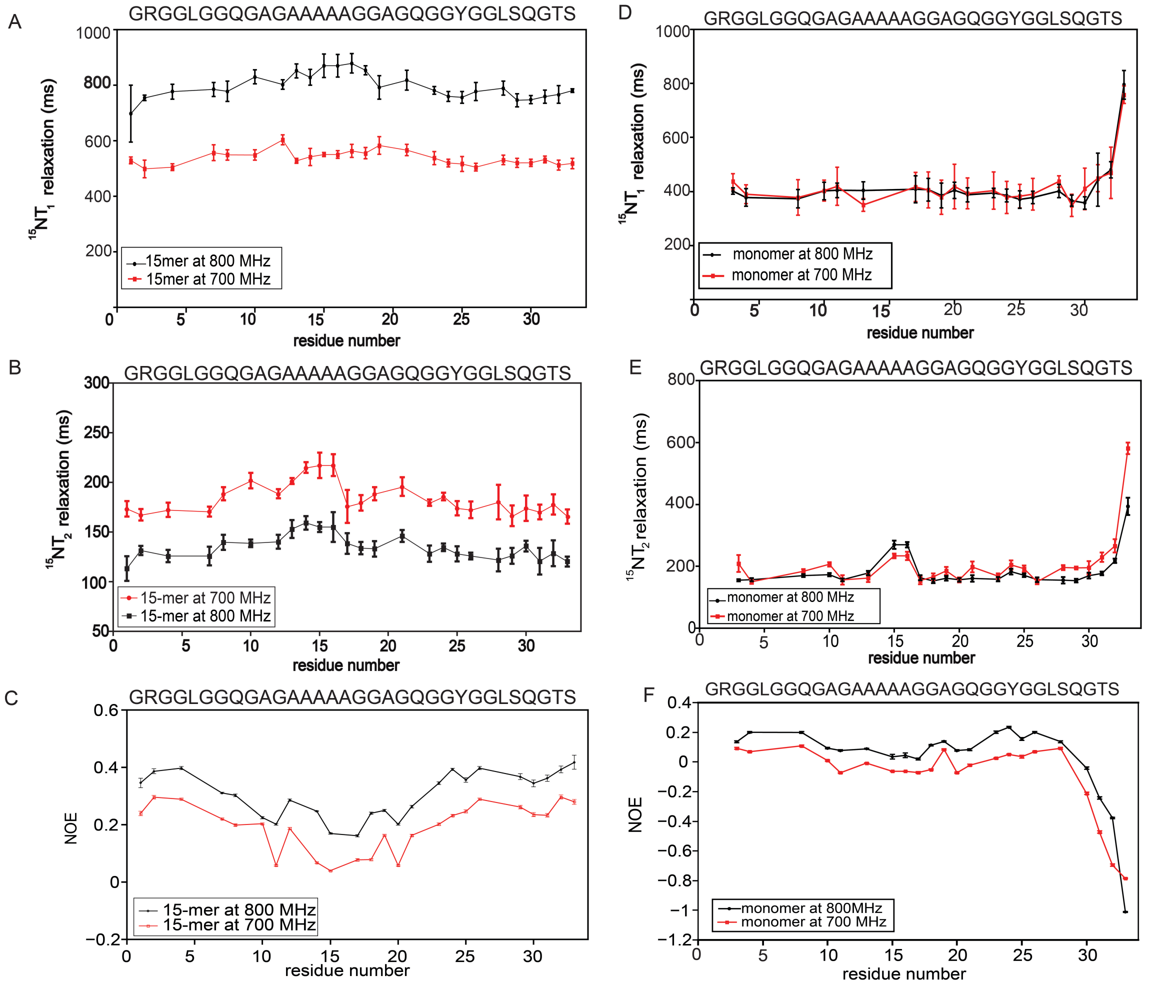

Supplementary Figure 7: Backbone ^{15}NT$_1$ and ^{15}NT$_2$ relaxation and {^{1}H}-^{15}N heteronuclear NOE of the 15-mer and monomer at pH 7, 10°C and two different magnetic fields: 700 MHz and 800 MHz. (A). Backbone ^{15}NT$_1$ relaxation of the 15-mer at pH 7, 10°C, 700 MHz (red) and 800 MHz (black). The average ^{15}NT$_1$ relaxation times of the 15-mer were 538 and 794 ms at 700 and 800 MHz, respectively (B). Backbone ^{15}NT$_2$ relaxation of the 15-mer at pH 7, 10°C, 700 MHz (red) and 800 MHz (black). The average ^{15}NT$_2$ relaxation times of the 15-mer at 700 and 800 MHz were 183 and 134 ms, respectively (C). {^{1}H}-^{15}N heteronuclear NOE of 15-mer at pH 7, 10°C and 700 MHz (red) and 800 MHz (black). {^{1}H}-^{15}N heteronuclear NOE is useful to probe the local dynamics (H-N bond vector) (D) Backbone ^{15}NT$_1$ relaxation of the monomer at pH 7, 10°C, 700 MHz (red) and 800 MHz (black). The average ^{15}NT$_1$ relaxation times of the monomer were 416 and 420 ms at 700 and 800 MHz (E) Backbone ^{15}NT$_2$ relaxation of the monomer at pH 7, 10°C, 700 MHz (red) and 800 MHz (black). The average ^{15}NT$_2$ relaxation times of the monomer (189 and 169 ms at 700 and 800 MHz, respectively (F) {^{1}H}-^{15}N heteronuclear NOE of the monomer at pH 7, 10°C and 700 MHz (red) and 800 MHz (black). Error bars in ^{15}NT$_1$ and ^{15}NT$_2$ relaxation were obtained from the confidence intervals calculated by Sparky, using the standard deviation of the data from the fitted line as an estimate for the standard deviation in intensity. Uncertainties in the {^{1}H}-^{15}N Heteronuclear NOE values were estimated from the base plane noise in the {^{1}H, ^{15}N}-HSQC spectra with and without proton saturation

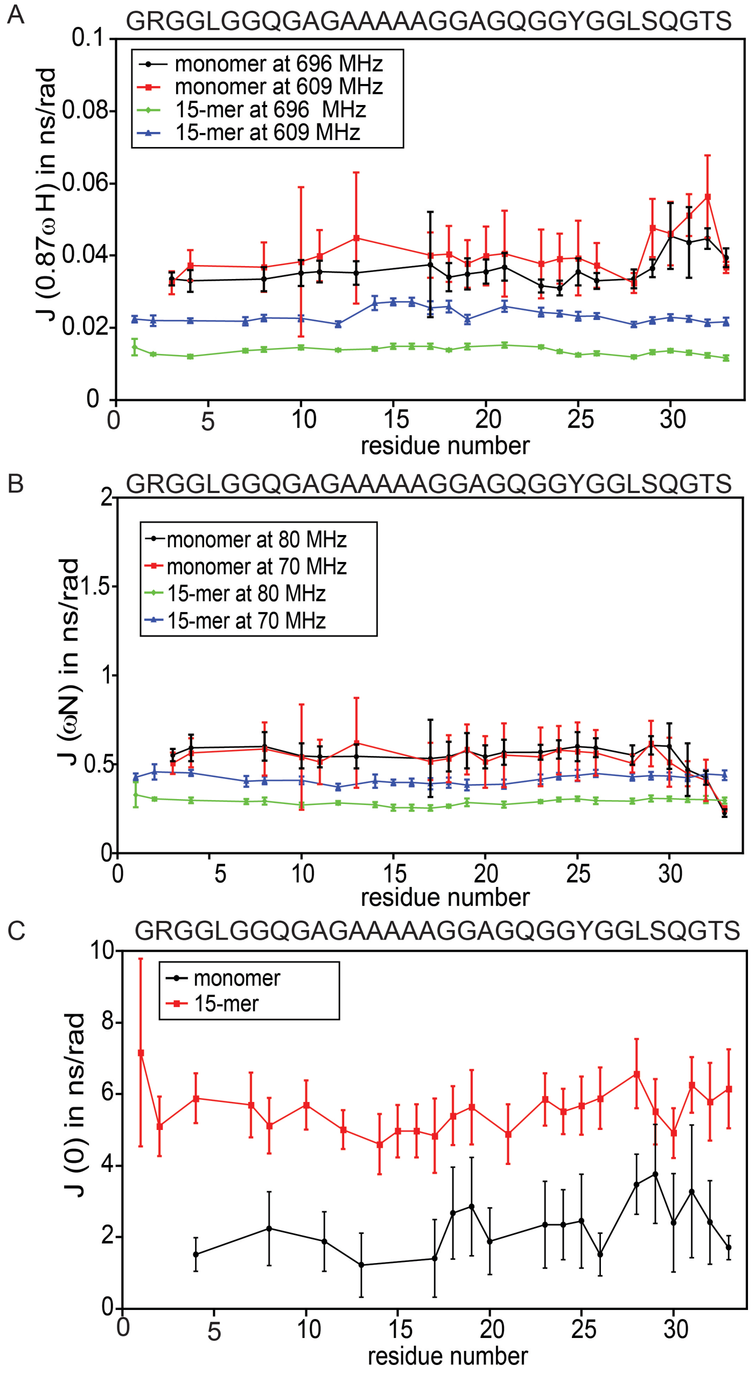

Supplementary Figure 8: **Spectral densities J(0.87 ωH), J(ωN) and J(0) of the 15-mer and monomer from 2 magnetic fields, 700 and 800 MHz.** (A) Spectral densities J(0.87 ωH) of the monomer and 15-mer at 700 and 800 MHz, which are represented by J(609 MHz) and J(696 MHz), respectively. The spectral density J(696 MHz) of the monomer is indicated in black, the spectral density J(609 MHz) of the monomer is indicated in red, the spectral density J(696 MHz) of the 15-mer is indicated in green, and the spectral density J(609 MHz) of the 15-mer is indicated in blue. The spectral densities J(609 MHz) and J(696 MHz) of the monomer were longer (~0.03 -0.04 ns/rad) than those of the 15-mer (~0.01-0.02 ns/rad), which indicates that monomer underwent faster motion than the 15-mer (B) Spectral densities J(ωN) of the monomer and 15-mer at 700 and 800 MHz, which are represented by J(70 MHz) and J(80 MHz), respectively. The spectral density J(80 MHz) of the monomer is indicated in black, the spectral

density J(70 MHz) of the monomer is indicated in red, the spectral density J(80 MHz) of the 15-mer is indicated in green, and the spectral density J(70 MHz) of the 15-mer is indicated in blue. The spectral densities J(70 MHz) and J(80 MHz) of the monomer were not significantly different, whereas the spectral density J(80 MHz) of the 15-mer decreased compared to its spectral density at J(70 MHz) (Supplementary Fig. 8B), which indicates that the monomer underwent faster motion than the 15-mer (C) Spectral density J(0) of the 15-mer (black) and monomer (red). Error bars of spectral densities were calculated based on the weighted sum of uncertainties from ^{15}N T_1, $^{15}NT_2$ and $\{^1H\}$-^{15}N Heteronuclear NOE[1].

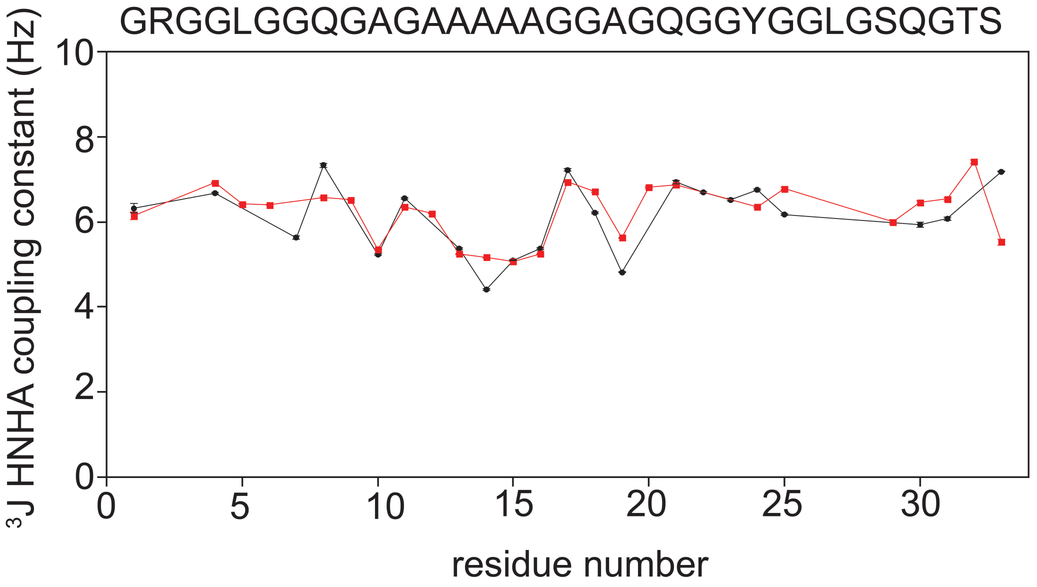

Supplementary Figure 9: ^{3}J HNHα coupling constant of the repetitive domain as a function of the residue number. Red indicates a ^{3}J HNHα coupling constant of the 15-mer, and black indicates a ^{3}J HNHα coupling constant of the monomer. For the 15-mer, the average ^{3}J HNHA coupling constant was 5.6 (s.d ±0.5) Hz in the polyalanine region and 6.5 (s.d±0.5) Hz in the glycine-rich region. For the monomer, the average ^{3}J HNHA coupling constant was 5.4(s.d.±0.4 Hz in the polyalanine region and 6.4±0.7 Hz in the glycine-rich region. ^{3}J HNHα coupling constant data from overlapping peaks were not included in the graph. Error bar for each point in the coupling constant was calculated from the peak intensities and base plane noise levels from 3D HNHA spectrum.

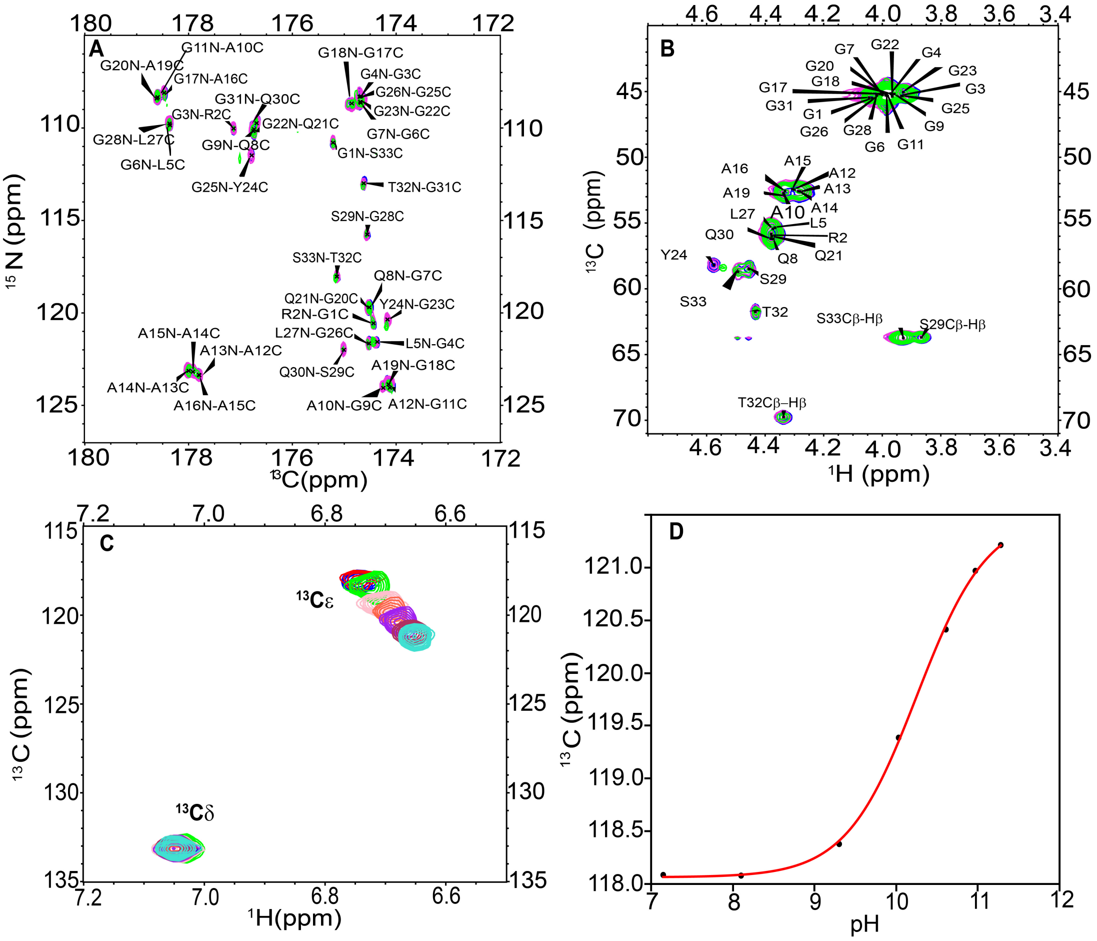

Supplementary Figure 10: Effect of pH on the repetitive domain (15-mer) in the basic pH range. (A) Overlay of the 2D CON spectra of the 15-mer at pH 7 (blue), pH 9 (magenta) and pH 10.2 (green). (B) Overlay of the 2D ^{1}H-^{13}C HSQC aliphatic spectra of the 15-mer at pH 7 (blue), pH 9 (magenta) and pH 10.2 (green). (C) Chemical shift changes in the $^{13}C\varepsilon$ Tyr aromatic ring upon pH titration (from pH 7–11.28). (D) pH titration profile of Tyr in the repetitive domain (15-mer) in 10 mM phosphate buffer at pH 7 and 10°C.

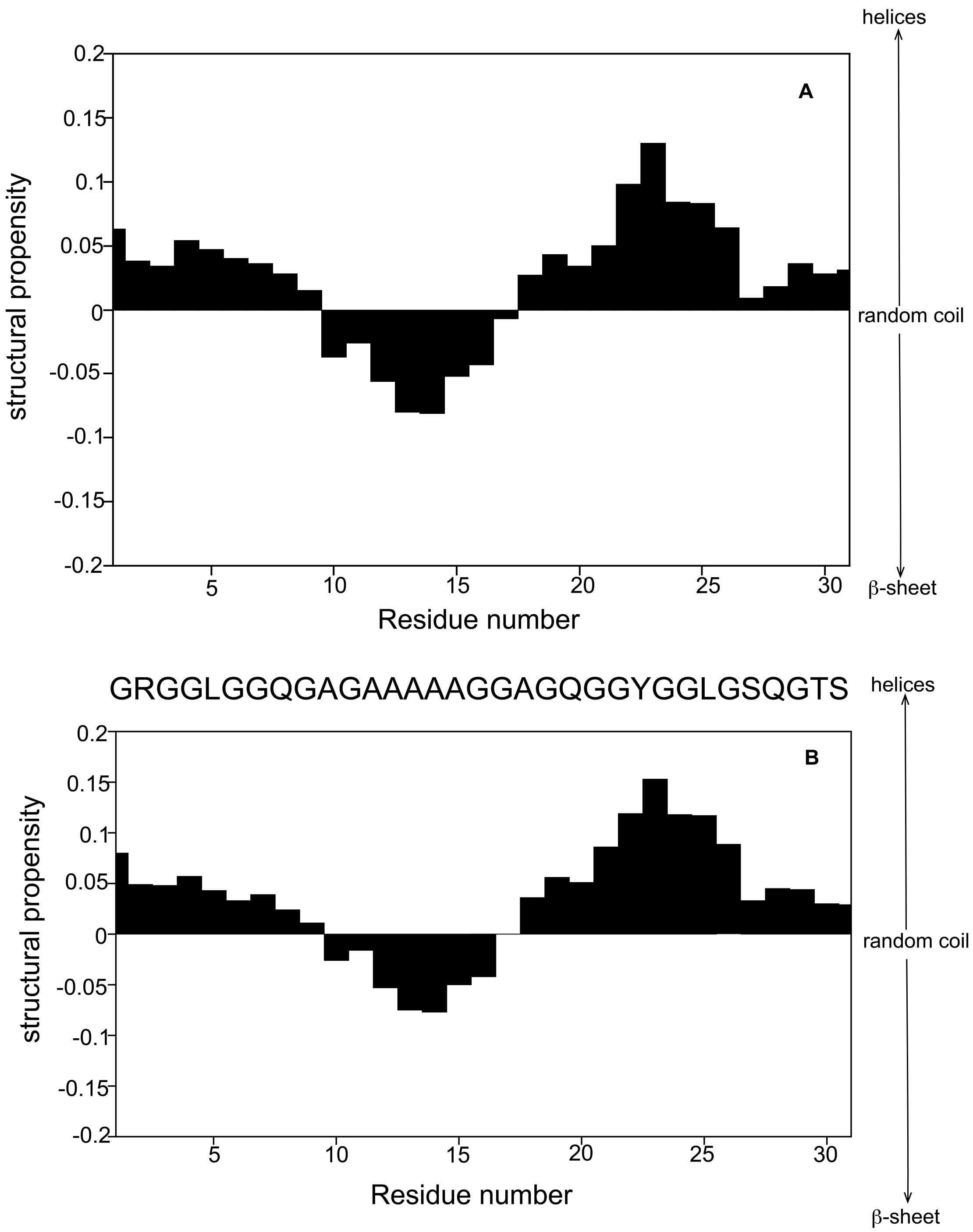

Supplementary Figure 11: Structural propensities of the 15-mer at (A) pH 7 and (B) 10.2. At pH 10.2, the 15-mer displays a slight increase in helical propensity near Y24.

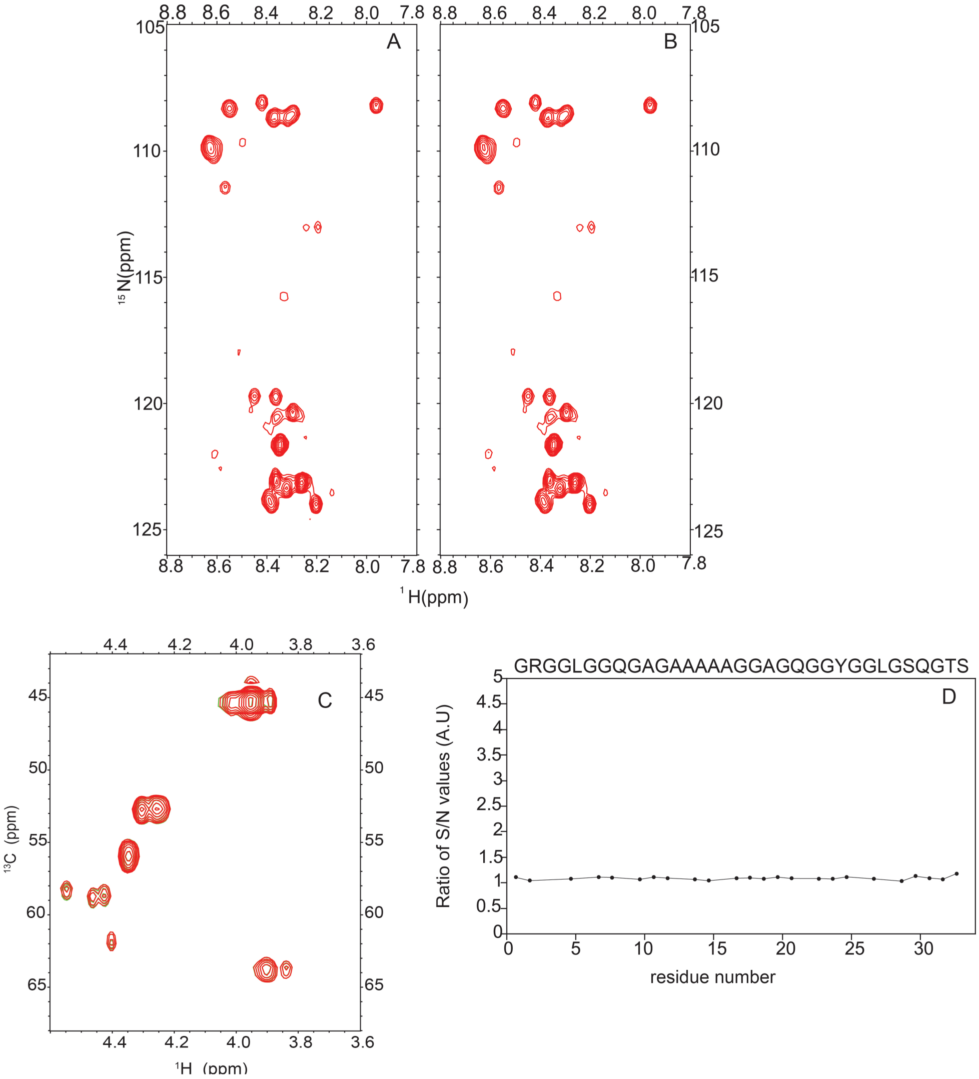

Supplementary Figure 12: Concentration effect on the repetitive domain. (A) ^{1}H-^{15}N HSQC spectra of the 15-mer without dilution (1 mM); number of scans=4. (B) For two-fold dilutions (0.5 mM), the number of scans=16. (C) Overlay of the Cα-Hα region of aliphatic ^{1}H-^{13}C HSQC of the 15-mer at different concentrations. Green is without dilution (concentration=1 mM, number of scans=4), and red is with two-fold dilutions (concentration=0.5 mM, number of scans=16). (D) Signal-to noise (S/N) ratio values of ^{1}H-^{15}N HSQC spectra of the 15-mer (concentration 1 mM, number of scans=4) and the 15-mer (concentration 0.5 mM, number of scans=16) as a function of the residue number. All spectra are processed in the same manner, and some overlapping peaks are not included in this graph.

A more detailed explanation of the signal-to-noise ratio of protein at different concentrations (**Supplementary Fig. 12D**) is provided as follows.

The signal-to-noise (S/N) value is proportional to the square root of the number of scans ($\sqrt{N}$) and concentration of the NMR samples[1].

$$S/N \sim conc \sim \sqrt{N}$$

Assuming that no change occurs in the interaction or dynamics of protein at different concentrations, to obtain the same signal-to-noise ratio, when the concentration B is ½ concentration A, then the number of scans of B should be 4 times the number of scans of A. The S/N ratio of the signals from the 2D ^{1}H-^{15}N HSQC spectrum of the 15-mer with two serial dilutions (16 scans) to the 15-mer without dilution (4 scans) was approximately one and thus

was uniform for the entire sequence. This result suggested that no changes were observed in the interactions or dynamics of the repetitive domain at higher concentrations.

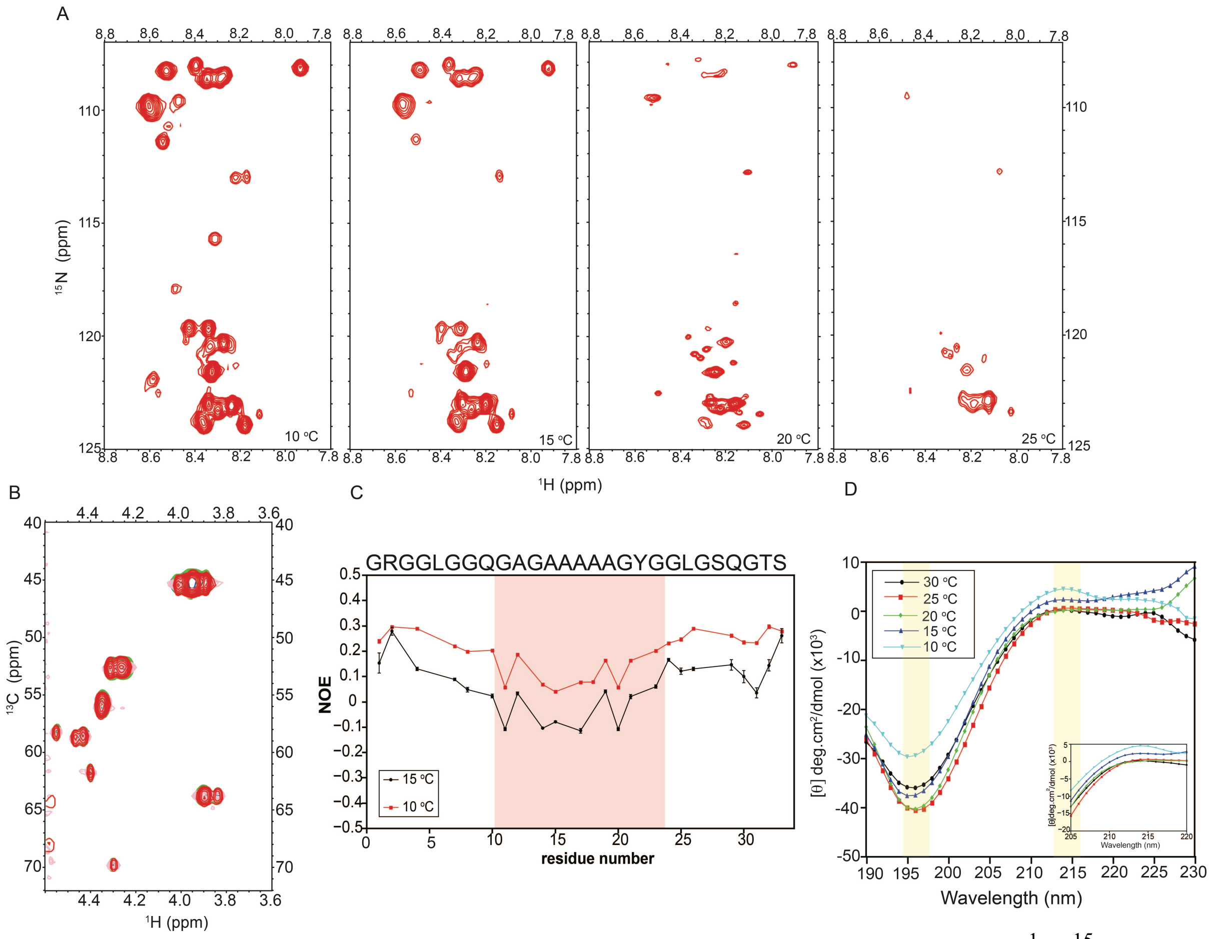

Supplementary Figure 13: Temperature effect on the repetitive domain. (A) ^{1}H-^{15}N HSQC spectra of the 15-mer from 10°C until 25°C. As the temperature increased, the signal intensities of the amide proton of the 15-mer decreased due to the rapid exchange of the amide proton signal with water. (B) Overlay of Cα- Hα region of ^{1}H-^{13}C-HSQC of the 15-mer at different temperatures. Blue is 10°C, green is 15°C, pink is 20°C and red is 25°C. No changes of Cα- Hα chemical shifts of 15-mer at different temperatures were observed. This finding indicates that conformational changes of the 15-mer do not occur at different temperatures. (C) {^{1}H}-^{15}N heteronuclear NOE of the 15-mer at 10°C and 15°C. The glycine-rich region consistently displayed limited flexibility compared with the polyalanine region (in red shadow) at different temperatures. (D) CD spectra of the 15-mer from 10°C up to 30°C. CD spectra of the 15-mer at different temperatures always showed minima at 196 nm and weak positive maxima at approximately 215 nm, which indicates that the 15-mer always contains PPII helix populations at different temperatures. However, as shown in the insert of Fig. 13D, the ellipticity of the 15-mer near wavelength 215 nm decreased at higher temperatures, which suggested that the PPII helix population decreased when the temperature increased.

Nephila clavipes major ampullate spidroin 1 (M37137.2)

```
GA
GAAAAAA  GGAGQGGYGGLGGQGAGQGGYGGLGGQGA GQGA
GAAAAAAA GGAGQGGYGGLGSQGAGR GGQGA
GAAAAAA  GGAGQGGYGGLGSQGAGR GGLGGQGA
GAAAAAAA GGAGQGGYGGLGNQGAGR GGQ
GAAAAAA  GGAGQGGYGGLGSQGAGR GGLGGQGA
GAAAAAA  GGAGQGGYGGLGGQGAGQGGYGGLGSQGAGRGGLGGQGA
GAAAAAAA GGAGQ    GGLGGQGAGQGA
GASAAAA  GGAGQGGYGGLGSQGAGR GGEGA
GAAAAAA  GGAGQGGYGGLGGQGAGQGGYGGLGSQGAGRGGLGGQGA
GAAAA    GGAGQ    GGLGGQGAGQGA
GAAAAAA  GGAGQGGYGGLGSQGAGR GGLGGQGA
GAVAAAAA GGAGQGGYGGLGSQGAGR GGQGA
GAAAAAA  GGAGQRGYGGLGNQGAGR GGLGGQGA
GAAAAAAA GGAGQGGYGGLGNQGAGR GGQ
GAAAAA   GGAGQGGYGGLGSQGAGR GGQGA
GAAAAAAV  GAGQEGIRGQ    GAGQGGYGGLGSQGSGR GGLGGQGA
GAAAAAA  GGAGQ    GGLGGQGA    GQGA
GAAAAAA  GGVRQGGYGGLGSQGAGR GGQGA
GAAAAAA  GGAGQGGYGGLGGQGVGR GGLGGQ
GAGAAAA  GGAGQGGYGGVGSGA
```

Nephila inaurata madagascariensis major ampullate spidroin 1 (AAK30606.1)

```
GLGGQGAGQ
GAGAAAAAA  GGAGQGGYGGLGSQGAGRGGYGGQ
GAGAAAAAAA GGAGQGGYGGLGSQGAGQGGYGGLGGQGAGQ
GAAAAAAA   GGAGQGGYGGLGSQGAGRGGYGGQ
GAGAAAAAT GGAGQGGYGGVGS
```

Nephila senegalensis major ampullate spidroin (AAK30608.1)

```
GLGGQGA
GRGAGAAAAAA GGAGQGGY GGLGGQGA
GAAAAAA     GGAGQGGQG  LGGRGAAAAGGAGQGGYGGLGGQGA
GRGAGAAAAAA GGAGQGGYGG LGGQGA
GAAAAAAAA   GGAGQGGYGG LGSQGAGRGGYGGQGA
GAAVAAI     GGVGQGGYGGVGSGA
```

Nephila antipodiana major ampullate fibroin 1 (ABC72644.1)

```
GGQGAGRGGYGGQGA
GAGAAAAAAA GGAGQGGYGGLGGQGAGQGGLGGQRA
GAAAAAA    GGAGQGGYGGLGSQGAGRGGYGGVGS
```

Nephila pilipes dragline silk spidroin 1 (AAV48948.1)

```
        GGAGQGGYGGLGGQGA
GAAGAGA    GQGGYGGLSGQGA
GAAAAAAGGA GQGGYGGLGGQGS
GAAAAGT    GQGGYGGLGGQGA
GAAGAAAAAVGGAGQGGYGGVGS
```

Nephilengys cruentata major ampullate spidroin-like protein (ABR37275.1)

```
GA
GAAAAAAAA GGAGQGGYGGLGGQGA
GAAAAAA   GGAGQGGYGGQ   GAGQ
GAAAAAA   SGAGQGGYEGP   GAGQGA
GAAAAAA   GGAGQGGYGGLGGQGAGQGA
GAAAAAA   GGAGQGGYGGLGGQGAGQGA
GAAAAAA   GGAGQGGYGGQ   GAGQ
GAAAAAA   GGAGQGGYGGLGSGQGGYGRQGA
GAAAAAAAA GGAGQGGYGGLGGQGA
GAAAAAA   GGAGQGGYGGQ   GAGQ
GAAAAAA   SGAGQGGYGGP   GAGQGA
GAAAAAA   GGAGQGGYGGLGGQGAGQGA
GAAAAAA   GGAGQGGYGGQ   GAGQ
GAAAAAA   GGAGQGGYGGLGSGQGGYGGQGA
GAAAAA    GGAGQGGYGGLGGQGAGQGA
GAAAAAA   GGSGRGGYGSQ   GAGQ
GAAAAAA GGAGQGGYGGAGS
```

Supplementary Note 1: Amino acid sequences of the repetitive domain of major ampullate spidroin 1 from the *Nephilidae* family. LGGQG, LGGSQG and LGNQG are shown in red, and the polyalanine regions are indicated in blue. GenBank accession codes are indicated with the protein names.

Supplementary Reference

1. Peng, J. W. & Wagner, G. Mapping of spectral density functions using heteronuclear NMR relaxation measurements. *J. Magn. Reson. 1969* **98,** 308–332 (1992).
2. Hoult, D. I. & Richards, R. E. The signal-to-noise ratio of the nuclear magnetic resonance experiment. *J. Magn. Reson.* **24,** 71–85 (1976).
